# “It is this very knowledge that makes us doctors”: an applied thematic analysis of how medical students perceive the relevance of biomedical science knowledge to clinical medicine

**DOI:** 10.1186/s12909-020-02251-w

**Published:** 2020-10-12

**Authors:** Bonny L. Dickinson, Kristine Gibson, Kristi VanDerKolk, Jeffrey Greene, Claudia A. Rosu, Deborah D. Navedo, Kirsten A. Porter-Stransky, Lisa E. Graves

**Affiliations:** 1 Department of Biomedical Sciences, Mercer University School of Medicine, Macon, GA USA; 2 Department of Family and Community Medicine, Western Michigan University Homer Stryker M.D. School of Medicine, Kalamazoo, MI USA; 3 Center for Interprofessional Studies and Innovation at Massachusetts General Hospital Institute of Health Professions, Boston, MA USA; 4 Massachusetts General Hospital, Learning Laboratory, Massachusetts General Hospital, Boston, MA USA; 5 Department of Biomedical Sciences, Western Michigan University Homer Stryker M.D. School of Medicine, Kalamazoo, MI USA

**Keywords:** Adaptive expertise, Professional identity formation, Biomedical science knowledge, Applied thematic analysis, Medical students, Undergraduate medical education, Basic science knowledge, Lifelong learning

## Abstract

**Background:**

Intensive study of the biomedical sciences remains a core component of undergraduate medical education with medical students often completing up to 2 years of biomedical science training prior to entering clerkships. While it is generally accepted that biomedical science knowledge is essential for clinical practice because it forms the basis of clinical reasoning and decision-making, whether medical students perceive an expanded role for their biomedical science knowledge remains to be examined.

**Methods:**

We conducted a qualitative research study to explore how medical students in the first clerkship year perceived the relevance of biomedical science knowledge to clinical medicine during this pivotal time as they begin their transition from students to physicians. To identify previously unidentified perspectives and insights, we asked students to write brief essays in response to the prompt: How is biomedical science knowledge relevant to clinical medicine? Ten codes and four themes were interpreted through an applied thematic analysis of students’ essays.

**Results:**

Analysis of students’ essays revealed novel perspectives previously unidentified by survey studies and focus groups. Specifically, students perceived their biomedical science knowledge as contributory to the development of adaptive expertise and professional identity formation, both viewed as essential developmental milestones for medical students.

**Conclusions:**

The results of this study have important implications for ongoing curricular reform efforts to improve the structure, content, delivery, and assessment of the undergraduate medical curriculum. Identifying the explicit and tacit elements of the formal, informal, and hidden curriculum that enable biomedical science knowledge to contribute to the development of adaptive expertise and professional identity formation will enable the purposeful design of innovations to support the acquisition of these critical educational outcomes.

## Background

Medical students often complete up to 2 years of intensive study in the biomedical sciences in the undergraduate medical curriculum. This knowledge base serves as the foundation for clinical reasoning and decision-making, and is required to address novel, complex, and ambiguous clinical problems that necessitate a deeper fund of knowledge, one that goes beyond reliance on pattern recognition and algorithms alone [1–6]. A detailed understanding of the biomedical sciences also enables physicians to understand and effectively utilize innovations and discoveries that emerge from basic and translational science research [7–9]. Thus, although there is general agreement that biomedical science knowledge is critical to the training of future clinicians [3, 10–16], debate remains about the depth and extent of training required [1, 17–19]. This debate is particularly relevant given continued efforts to reduce the time of training to address physician shortages, reduce the rising cost of physician training, and enable creation of time variable flexible and individualized learning pathways [20–23].

Innovative approaches to reform undergraduate medical education over the past 10 years include changes to curricular structure/organization, content, and delivery [23, 24]. Such innovations have the potential to significantly impact how the biomedical sciences are taught and learned. For example, many schools have embraced a reduction in the length of the preclinical curriculum and the inclusion of early clinical experiences in the preclinical curriculum where the majority of the biomedical sciences is taught [20, 21, 24]. These innovations may compress an already crowded curriculum in which contemporary topics are also being added such as health systems science, addiction, pain management, population health, social determinants of health, wellness, and medical informatics to name a few [24]. While evidence suggests that some of these changes may not impact academic performance per se [25], whether these and other critical outcomes of medical education such as professionalism, professional identity formation, adaptive expertise, and humanistic approaches to patient care are impacted remains to be examined [26]. For these reasons, further study is needed to anticipate how curricular reforms that impact training in the biomedical sciences might influence physician training.

To date, few studies have sought to understand medical student perceptions of their training in the biomedical sciences. Filling this gap in our understanding could lend significant insight to the question of the depth, context, and extent of training required in the biomedical sciences to ensure the effectiveness of initiatives to improve medical education. In one study, for example, the longitudinal development of students’ attitudes concerning the basic sciences revealed that students further in their training were more likely than beginning students to support learning biomedical knowledge prior to its application in a clinical context [27]. These results suggest that the experiences of more advanced students improved their recognition that knowledge of the biomedical sciences is important for medical practice. A more recent mixed methods study found that while most medical students agreed the biomedical sciences curriculum was a crucial part of their training, their perception of the importance and relevance of the biomedical curriculum decreased with their progress in medical school, which contradicts the results of the aforementioned study [28].

We conducted a qualitative research study to determine whether medical students perceive an expanded role for the biomedical science knowledge they acquire during training beyond those roles previously identified by surveys, open-ended questions, and focus groups. The objective this study was to explore how medical students in their first clerkship year perceive the relevance of biomedical science knowledge to clinical medicine with the goal of providing insights relevant to curricular reform efforts that impact how the biomedical sciences are taught.

## Methods

### Qualitative approach

We conducted an applied thematic analysis of participants’ essays. Applied thematic analysis is a rigorous, inductive set of procedures designed to identify and examine themes from textual data [29].

### Participants

This study included all fifty-five third-year medical students enrolled in the graduating class of 2019 (the second class enrolled since inception of the school). Twenty-six women and 29 men comprised the class of 2019.

### Context

This study took place over a 12-month period at the Western Michigan University Homer Stryker M.D. School of Medicine, a private not-for-profit graduate entry medical school. The preclinical curriculum has been previously described [30]. Briefly, at the beginning of the first year, students complete a medical first responder course and are licensed in the State of Michigan as Medical First Responders. Then, students learn fundamental concepts in the basic sciences in five Foundations of Health and Disease courses that range from three to 5 weeks in duration: Molecular, Cellular, Genetic, Metabolic, and Immunology and Infectious Disease. These foundational concepts are then revisited with increasing complexity throughout the remaining preclinical curriculum organized as nine organ-based courses ranging from five to 6 weeks in duration and which follow a multidisciplinary integration model: Hematology and Oncology, Musculoskeletal System and Dermatology, Cardiovascular System, Pulmonary System, Renal and Genitourinary System, Gastrointestinal System, Endocrinology and Reproduction System, Nervous System I, and Nervous System II. Foundational and organ-based courses were designed and delivered by both basic science and clinician educators to emphasize integration of the biomedical and clinical sciences. Integration was supported by weekly Team-Based Learning® experiences that were designed and delivered by teams of basic science and clinical faculty [31]. Integration also occurred in other curricular events such as case-based learning, simulation-based learning, tutorials, and anatomy, histology, and pathology laboratories.

Concurrent clinical courses in the first 2 years provide students with clinical skills and interactions with real patients, standardized patients, and simulated patients. These courses include Introductory Clinical Experiences, Advances and Perspectives in Medicine, early electives, and Professions of Medicine. In the third year, four blocks of 12–13 students rotate through four clerkship experiences in different sequences: Medicine and Neurology, Pediatric and Adolescent Medicine and Family and Community Medicine, Surgery, and Women’s Health and Psychiatry. In the fourth year, students complete electives and advanced courses in Critical Care Medicine, Emergency Medicine, Hospital-Centered Medicine, and Advanced Ambulatory Medicine.

### Sampling strategy, data collection and analysis

A pre-clerkship assignment was provided at the beginning the family and community medicine and pediatric and adolescent medicine clerkship block (Additional file 1). Students were asked to write a brief essay in response to the prompt: How is biomedical science knowledge relevant to clinical medicine? After completing the pre-clerkship assignment, a mid-clerkship assignment was provided to students at the beginning of the second half of the rotation (Additional file 1). This reflective writing assignment was designed using the principles of the Kolb experiential learning model [32]. Using this framework, students were asked to: 1) select a patient encounter (concrete experience), 2) identify and fill gaps in biomedical science knowledge (reflective observation), 3) reflect on how the new learning impacted the care of their patient (abstract conceptualization), and 4) consider how this process may impact their future clinical practice, and reevaluate their perception of the relevance of biomedical science knowledge to clinical medicine (active experimentation). An essay format for the assignments was selected to facilitate the collection of textual data from all 55 students to provide a deep understanding of students’ perceptions. The assignments were completed by all 55 students as part of the required instructional elements in the clerkship rotation. The assignments were designed to be contributory to the final grade for the clerkship but were not a deciding factor in passing the clerkship.

Prior to beginning the data analysis, participant essays were collected and de-identified. Four members of the research team (B.L.D., J.G., K.A.P.-S. and L.G.), who were not involved in assessing student performance in this clerkship, read through all 55 participants’ essays. Two essays were eliminated from the study because they were not responsive to the pre-clerkship prompt, leaving a total of 53 essays for analysis. An applied thematic analysis of participant essays was conducted following the procedures described by Braun and Clark to identify codes and themes from qualitative data [33]. Following manual open coding, a codebook was created through consensus discussion during research team meetings. Once the codebook was created, codes were synthesized into themes and all essays were analyzed using ATLAS.ti for data organization and retrieval (ATLAS.ti Scientific Software Development GmbH). Student quotes selected to illustrate themes in the Results section are followed by a tag in parentheses to indicate the rotation block [1–4] and student (A-Y).

To increase the validity of our findings, the research team used the verification procedures of maintaining an audit trail, discussing our own biases to promote reflexivity, describing in detail how data were collected and analyzed, relating the findings to the existing literature, analyzing the data in a systematic manner, and involving more than one person in the analysis, which included faculty from a range of backgrounds: two biomedical science faculty members (B.L.D. and K.A.P.-S.), a member of the department of medical education (J.G.), three clinician educators (K.G., L.G., and K.V.), and two faculty with expertise in qualitative research (D.D.N. and C.A.R.). Finally, intensity sampling was selected as a purposeful sampling strategy to identify four study participants for a focus group that was held at the conclusion of the clerkship rotations as a method of member checking [34]. This sampling strategy was selected to gather information-rich perspectives of the phenomenon under study.

## Results

To determine whether medical students entering the first year of clerkships perceive an expanded role for their biomedical science knowledge beyond its role in clinical reasoning and decision making, we asked students at the beginning of their family and community medicine and pediatric and adolescent medicine clerkship to reflect on how biomedical science knowledge was relevant to clinical medicine. Open coding identified 10 codes that were synthesized into four themes that described medical students’ perceptions: knowledge to practice medicine, lifelong learning, physician-patient relationship, and learner perception of self (Table 1). We next examined students’ responses to prompts in the mid-clerkship reflective writing assignment to identify support for the codes and themes. Specifically, students were asked to: 1) select and briefly describe a patient encounter, 2) think about the patient’s illness or disease process through a basic science lens by accessing, exploring, and extending their biomedical science knowledge, 3) reflect on how the new learning impacted the care of their patient, and 4) consider how this process may impact their future clinical practice, and reevaluate their perception of the relevance of biomedical science knowledge to clinical medicine. Below, we describe the codes and themes and provide representative student quotes.

**Table 1:** Themes, theme definitions, and codes of medical students’ essays

Themes	Theme definitions	Codes
**1. Knowledge to practice medicine**	Use biomedical science knowledge to support clinical reasoning and justify clinical decisions	1.a. Diagnosis 1.b. Patient management 1.c. Tolerance of ambiguity 1.d. Patient safety
**2. Lifelong learning**	Acquire new biomedical science knowledge to understand and apply new, improved, and emerging therapies/treatments, diagnostics, interventions, and understanding of disease mechanisms that are advanced through biomedical science research (i.e., evidence-based medicine)	2.a. Continue learning throughout practice
**3. Physician-patient relationship**	Educate and empower patients by engaging them in shared decision making, providing compassionate care, and developing patient trust	3.a. Educate patients 3.b. Empower patients 3.c. Develop patient trust
**4. Learner perception of self**	Biomedical science knowledge contributes to the emergence of professional identity	4.a. Develop confidence and competence as a physician 4.b. Transition from layperson to physician

### Theme 1: knowledge to practice medicine

This theme developed from the clustering of four codes: diagnosis, patient management, tolerance of ambiguity, and patient safety (Table 1), and addresses the use of biomedical science knowledge to support clinical reasoning and to justify clinical decisions.

#### Code 1a: diagnosis

This code included many of the physician tasks required to formulate a diagnosis (Table 2) and had an overall frequency of 30% (Table 3).

**Table 2:** Codes and code definitions

Codes	Code definitions
**1.a. Diagnosis**	Conduct physical exam, interview patient and collect history of present illness, recognize signs and symptoms, link symptoms to disease processes, assess risk factors for disease, form initial impressions, formulate a testable hypothesis, create a differential diagnosis, select diagnostic studies and labs, interpret diagnostic data, identify likely etiology
**1.b. Patient management**	Select treatment/therapy, understand treatment mechanism of action, understand treatment limitations, identify treatment contraindications, plan for short and long term clinical management, patient follow-up, anticipate/recognize course of illness, anticipate worsening condition, anticipate disease outcomes/prognosis, anticipate longitudinal changes to health, anticipate future healthcare needs, make recommendations for preventative care, anticipate side effects/complications of treatment, optimize treatment plans
**1.c. Tolerance of ambiguity**	Recognize patient variability with respect to disease, anticipate complications of disease, recognize comorbidities influencing outcomes, tolerate unusual, novel, complex or ambiguous cases, recognize patient variability with respect to response to treatment, recognize comorbidities influencing treatment, anticipate and recognize confounding factors or variables contributing to disease
**1.d. Patient safety**	Prevent a missed opportunity for early treatment/intervention in disease, prevent misdiagnosis, prevent medical errors, avoid use of inappropriate treatments
**2.a. Continue learning throughout practice**	Continue to acquire new knowledge by engaging with the literature to understand advances in science and medicine, continue medical education and training throughout practice, critically evaluate the research literature to apply new and emerging diagnostics, therapies, interventions, personalized medicine, approaches for prophylaxis, and approaches for disease prevention/preventative medicine to patient care
**3.a. Educate patients**	Answer patient questions and respond to patient concerns, dispel incorrect medical information, destigmatize misconceptions of disease, explain disease and treatments in terms that are understandable to patients
**3.b. Empower patients**	Advocate for lifestyle changes, empower patients to become actively involved in their own health and health maintenance, engage patients in share decision making regarding their health and various treatment options
**3.c. Develop patient trust**	Patients expect physicians to have a wealth of biomedical science knowledge and this contributes to development of patient trust and provides the foundation for empathetic and compassionate care
**4.a. Develop confidence and competence as a physician**	Knowledge of biomedical science contributes to the development of confidence and competence as a physician, biomedical science knowledge provides a common language used to engage with colleagues and other members of the healthcare team
**4.b. Transition from layperson to physician**	Knowledge of biomedical science is expected of oneself, one’s colleagues, and patients and thus forms the basis of one’s emerging role as a physician

**Table 3:** Code frequencies

Codes	Block 1	Block 2	Block 3	Block 4	Total
1.b. Patient management	9 (17%)	10 (19%)	10 (19%)	9 (17%)	38 (72%)
2.a. Continue learning throughout practice	7 (13%)	5 (9%)	9 (17%)	8 (15%)	29 (55%)
1.a. Diagnosis	8 (15%)	2 (4%)	3 (6%)	3 (6%)	16 (30%)
3.a. Educate patients	6 (11%)	3 (6%)	3 (6%)	3 (6%)	15 (28%)
1.c. Tolerance of ambiguity	5 (9%)	2 (4%)	3 (6%)	2 (4%)	12 (23%)
3.b. Empower patients	5 (9%)	1 (2%)	1 (2%)	6 (11%)	10 (19%)
1.d. Patient safety	3 (6%)	2 (4%)	2 (4%)	2 (4%)	9 (17%)
3.c. Develop patient trust	5 (9%)	1 (2%)	0 (0%)	1 (2%)	7 (13%)
4.b. Transition from layperson to physician	2 (4%)	2 (4%)	0 (0%)	3 (6%)	7 (13%)
4.a. Develop confidence and competence as a physician	1 (2%)	0 (0%)	0 (0%)	3 (6%)	4 (8%)

Pre-clerkship assignment: “Biomedical science knowledge is relevant to clinical medicine because it allows for a deeper understanding of the disease processes occurring in patients and helps us make more informed decisions for their care. If we have a firm grasp of physiology and pathology, we can conceptualize what is “normal” in our patients and how that normal has been interrupted by disease.” (4 T).

Mid-clerkship assignment: “By thinking about basic science and what mechanisms could be causing the patient’s symptoms, I was better able to come up with possible differential diagnoses.” (1Y).

#### Code 1b: patient management

This code relates to various physician tasks required for patient management (Table 2) and had the highest overall frequency (72%) (Table 3).

Pre-clerkship assignment: “… understanding the science behind our actions allows us to anticipate the outcomes of our treatment … Without the basic science knowledge to guide our practice, we would just blindly follow clinical guidelines … [it is] important to understand the science behind those guidelines so that we can adjust accordingly, and better treat patients that might not fit in to a defined set of rules.” (3P).

Mid-clerkship assignment: “On initial glance, a rash, abdominal pain, and hypertension seem seemingly unrelated. However, the basic science understanding of Henoch-shönlein purpura helped pull all of these components together. The process of reading about Henoch-shönlein purpura was fundamental to providing the best care possible for our patient.” (1B).

#### Code 1c: tolerance of ambiguity

Tolerance of ambiguity captured the concept that patients and disease processes are complex, and management requires the application and integration of basic science and clinical science knowledge to provide optimal care (Table 2). This code had an overall frequency of 23% (Table 3).

Pre-clerkship assignment: “… clinical medicine is full of patterns and puzzles. Three patients who all come in with a cough can have extremely diverse disease processes and therefore require unique treatments … a thorough history and physical must be combined with basic science knowledge to accurately diagnose a patient.” (1B).

Mid-clerkship assignment: “Incorporating basic science objectives when treating patients allows you to modify your care for situations when treating patients that are not “traditional” patients. You can better adjust your care when patients have multiple active disease processes and medications with potential interactions.” (2 W).

#### Code 1d: patient safety

This final code within theme 1 focused on the use of biomedical science knowledge to prevent medical errors (Table 2) and had a frequency of 17% (Table 3).

Pre-clerkship assignment: “Without biomedical science knowledge, it is possible to misdiagnose patients or continue to make recommendations or prescribe treatments that, at best, don’t work and, at worst, do more harm than good.” (2S).

Mid-clerkship assignment: “… this deeper understanding was critical to ensuring that we were drawing the appropriate labs/imaging and monitoring the necessary vitals to prevent or address any complication.” (1B).

Finally, three students presented minority arguments that disagreed with the theme “knowledge to practice medicine”, and expressed views that biomedical science knowledge has no role in some physician skills, including communication and interpersonal skills:

“… there is very little correlation between biomedical science knowledge and how great someone might be in the clinic... Yes, the baseline knowledge might be important, but clinical knowledge has more to do with your ability to talk to a patient like a human being and help them feel better.” (1R).

### Theme 2: lifelong learning

This second theme addressed the need for physicians to continue to expand their biomedical science knowledge throughout their careers. This theme developed from a single code: continue learning throughout practice to understand and apply advances in science and medicine (i.e., evidence-based medicine) (Table 1).

#### Code 2a: continue learning throughout practice

This code reflected the need for physicians to continue to acquire new knowledge by engaging with the research literature to understand and apply advances in science and medicine (Table 2). This code had an overall frequency of 55% (Table 3).

Pre-clerkship assignment: “As new research emerges within the medical field, clinicians must rely on the knowledge they gained in medical school to process and understand the literature... Without [a] solid foundation of biomedical education, physicians would not be capable of synthesizing and comprehending the new data.” (4B).

Mid-clerkship assignment: “As I continue in my medical education, I believe that tying basic science concepts into my clinical practice will not only help me to better understand disease processes that I encounter, but it will also allow me to better adapt to new management and treatment approaches, because I will understand the underlying processes being targeted.” (4C).

### Theme 3: physician-patient relationship

The third theme of physician-patient relationship described the use of biomedical science knowledge to educate and empower patients. This theme developed from the clustering of three codes: educate patients, empower patients, and develop patient trust (Table 1).

#### Code 3a: educate patients

The ability to educate patients requires the use of biomedical science knowledge to answer patient questions, respond to patient concerns, dispel incorrect medical information, destigmatize misconceptions of disease, and explain disease and treatments in terms that are understandable to patients (Table 2). This code had an overall frequency of 28% (Table 3).

Pre-clerkship assignment: “… understanding the basic science behind disease allows the physician to better explain to patients what is happening to them. This leads to a better physician-patient relationship.” (1Y).

Mid-clerkship assignment: “Ultimately, the process of viewing a patient through a basic science lens helps to educate oneself which, in turn, translates into an opportunity to educate the families and [the] rest of the medical team on rounds or presentations.” (1P).

#### Code 3b: empower patients

Students recognized the use of biomedical science knowledge to empower patients to become actively involved in their own healthcare (Table 2) and had a frequency of 19% (Table 3).

Pre-clerkship assignment: “A physician must have the knowledge, and the ability to accurately inform patients of their options, allowing the patient to make an informed decision about the course they choose to take, as well as the potential ramifications of that choice such as drug side effects, and the consequences of not taking a particular medication.” (4D).

Mid-clerkship assignment: “This not only equips me to better manage those conditions, but also to build better relationships with my patients. Furthermore, in educating patients about their conditions, they become more invested in their health and are better able to manage their medical problems as well.” (3P).

#### Code 3c: develop patient trust

This code captured the concept that patients (and society) expect physicians to have a wealth of biomedical science knowledge, and that this knowledge base contributes to establishing the trust of patients and providing compassionate care (Table 2). This code had an overall frequency of 13% (Table 3).

Pre-clerkship assignment: “Patients often want to know what is happening when they suffer from a disease and having the knowledge to explain this to them increases rapport and confirms the trust that they put in the physician. Patients want to feel like they are being cared for by an expert, and there is no better way to show expertise than to describe in detail what is happening, why it is happening, and what we can do to treat it effectively.” (4 T).

Mid-clerkship assignment: “Getting a new diagnosis can be confusing and overwhelming, and it can be reassuring when the doctor explains things. Even if he or she does not know exactly what is wrong, the effort to explain things on a level that the patient can understand can build rapport and be very important.” (2C).

### Theme 4: learner perception of self

The final theme, learner perception of self, captured how biomedical science knowledge contributes to the development of a professional identity. This theme developed from the clustering of two codes: develop confidence and competence as a physician and transition from layperson to physician (Table 1).

#### Code 4a: develop confidence and competence as a physician

This code was interpreted from student perceptions that biomedical science knowledge contributes to the development of confidence and competence as a physician and provides a common language in which to engage with colleagues and other members of the healthcare team (Table 2). This code had the lowest frequency (8%) (Table 3).

Pre-clerkship assignment: “When we understand *why* a certain treatment works, not just that it works, we develop true, long-lasting clinical knowledge that allows us to treat our patients with confidence. What’s more, the biomedical sciences are a language that we use to communicate with each other and with our research science colleagues.” (4S).

Mid-clerkship assignment: “The basic science preparation and reading really helped elevate the level of discussion when presenting to the attending.” (1B).

#### Code 4b: transition from layperson to physician

This code captured the idea that biomedical science knowledge is expected of oneself, one’s colleagues, and by one’s patients. Students perceived that the application of biomedical science knowledge in a clinical context forms the basis of their emerging identity as a physician (Table 2). This code had an overall frequency of 13% (Table 3).

Pre-clerkship assignment: “… if our knowledge were limited to asking a list of questions, identifying abnormalities, and prescribing the correct medications off a list of protocols then we would simply be technicians. Those skills can be programmed into a computer algorithm that can diagnose disease and treat patients. The value in doctors is that we are both clinicians and scientists.” (2F).

Mid-clerkship assignment: “And I think that must be a part of medical practice and education, to push those around us to continually fill the gaps in our knowledge, and remember the importance of the basics in leading to what we do.” (1A).

### Students’ learning trajectory

In analyzing the students’ essays in response to the pre-clerkship assignment prompt, we observed that the majority of responses reflected simplistic application of their biomedical science knowledge.

“Knowing that a drug only works when functioning beta cells are present allows me to recognize that these medications would not work for a type I diabetic because type 1 diabetics no longer have functioning beta cells.” (1 T).

Students’ responses reflecting a more sophisticated application of biomedical science knowledge were far fewer:

“Rather than memorizing what causes edema, I was able to use my basic science knowledge to not only know what might be causing it but why. This also helped me understand the treatment. For example, in the case of congestive heart failure, I knew that the cause of edema was increased hydrostatic pressure and the way to relieve that is to get rid of fluid. Therefore, I knew that using a diuretic in this case would help this person’s worsening edema.” (2A).

## Discussion

This study examined whether students perceived a role for their biomedical science knowledge beyond its previously articulated role in supporting clinical reasoning and decision making. To address this, we performed an applied thematic analysis of student essays in response to the prompt: How is biomedical science knowledge relevant to clinical medicine? The research team interpreted four themes through qualitative data analysis. Two themes, knowledge to practice medicine and lifelong learning relate to developing the skills and attributes of an expert physician. The remaining themes, physician-patient relationship and learner perception of self relate to the process of assuming an identity aligned with that of a physician. These findings suggest that students perceive a role for their biomedical science knowledge as contributory to the constructs of adaptive expertise and professional identity formation.

Ericsson defines expertise as ‘the characteristics, skills, and knowledge that differentiate experts from novices’ [35]. In medicine, adaptive expertise requires both efficiency, which is defined as the use of biomedical knowledge to solve routine problems, and innovation, in which knowledge is used to create new solutions to solve novel problems [36–38]. Professional identity formation refers to a student’s transformation from lay person to physician, and is recognized as a key transition in medical student training, requiring the student to integrate the knowledge, skills, values, and behaviors of a competent, humanistic physician with his or her own unique identity and core values [39–41].

The theoretical framework of adaptive expertise emphasizes flexibility and innovation in practice and requires a physician to make efficient use of their previously acquired knowledge to solve routine problems and also to create new knowledge when confronted with novel, non-routine problems [42]. The first theme, knowledge to practice medicine, suggests that students recognized that biomedical science knowledge is required for them to begin to develop the adaptive expertise that characterizes expert physicians. Student essays robustly addressed the efficiency dimension of adaptive expertise in which biomedical science knowledge was required to perform various aspects of patient care reflected in the first four codes: diagnosis, patient management, tolerance of ambiguity, and patient safety. That students perceived a role for their biomedical science knowledge in these physician tasks associated with the efficiency domain of adaptive expertise, but not the innovation phase, was anticipated; while expert clinicians are able to embrace complexity while acting with simplicity, novice learners struggle to embrace simplicity [13, 43]. This observation was also consistent with the finding that medical students in their third and fourth year of training are immersed in the efficiency dimension of adaptive expertise, but do not perceive that they have a role in innovation in practice, a key aspect of the innovation dimension of adaptive expertise [37].

Student responses also aligned with the first two of four key phases of the master adaptive learner conceptual model, which is a metacognitive approach based on self-regulation that fosters the development of adaptive expertise: the planning phase in which the learner identifies a gap in knowledge, skills or attitudes, and the learning phase in which the learner selects an opportunity for learning and searches for resources [38, 44]. Student essays reflected the early phases of the master adaptive learner in which gaps in biomedical science knowledge are identified and filled during patient encounters. The remaining phases of this model characterize more advanced learners: the assessing phase in which the learner tries out the new knowledge and assesses its effectiveness, and the adjusting phase in which new leaning becomes incorporated into everyday practice. Given that our students were in their third year of training, it was not surprising that evidence of these more advanced phases was lacking.

The second theme, lifelong learning, developed from students’ understanding that they must have a solid foundation of biomedical science knowledge to continue to learn throughout practice (the fifth code). Learning new biomedical science knowledge that is advanced through research is required to maintain and improve the physician’s ability to solve routine clinical problems and to begin to solve novel, complex, and unfamiliar problems, thus linking this knowledge as requisite to the development of adaptive expertise.

A key developmental milestone in medical student training is the transformation from lay person to physician through a process termed professional identity formation. Jarvis-Selinger et al. define professional identity formation as ‘An adaptive developmental process that happens simultaneously at two levels: 1) at the level of the individual, which involves the psychological development of the person and 2) at the collective level, which involves the socialization of the person into appropriate roles and forms of participation in the community’s work’ [45]. Professional identity formation is now viewed as an educational objective of medical education [22, 46]. The third and fourth themes identified in this study, physician-patient relationship and learner perception of self, capture key elements of this developmental process. The theme physician-patient relationship developed from three codes that suggest that a physician uses biomedical science knowledge to educate patients about a diagnosis or treatment plan, empowers patients to become active participants in their healthcare, and establishes trusting relationships with patients. These are the skills and behaviors of a competent physician and core elements of a physician’s professional identity. The fourth theme, learner perception of self, directly speaks to the role of biomedical science knowledge in the act of becoming a physician. This theme derived from two codes in which students recognized that their biomedical science knowledge base enables a sense of confidence and competence, and thus contributes to their transition from layperson to physician. The link between these two codes and professional identify formation are succinctly summarized by one of the students: “It is this very knowledge that makes us doctors.”

Professional identity formation is thought to be triggered by experiences of cognitive disequilibrium in relationship to students’ perceptions of self-in-profession, such as the transition from undergraduate student to medical student, early clinical experiences in the preclinical years, exposure to the business of medicine, and exposure to physicians in clinical practice, all of which represent vulnerable periods of training [47]. Students also expressed that biomedical science knowledge is required to competently communicate with colleagues and other members of the healthcare team, and that this knowledge provides the foundation for building confidence, credibility, and competence as a physician. That a solid foundational knowledge of biomedical science is a professional expectation was recognized by the students is congruent with other studies that identify this as a societal expectation [48, 49]. Whether our students’ experience of an integrated curriculum and early clinical experiences contributed to their perceptions to enable us to identify a previously unreported role for biomedical science knowledge in the development of adaptive expertise and professional identity needs further study.

### Study limitations

This study had a few limitations. First, the data were collected from an assessed assignment. We attempted to mitigate any potential bias by making the assignment low-stakes and assessed on a pass or fail scale such that it had no significant impact on the students’ overall clerkship grades. To further address this concern, a focus group was convened, and students were asked if they would have responded differently had the assignment been formative in nature. Students indicated that the essays might have less detail, but that the content would have remained the same. Whether or not the students’ responses to the assignment prompts represented their true perceptions or were influenced by the assignment, the students were still able to articulate links between their biomedical science knowledge and the practice and art of medicine without any explicit training in the concepts of adaptive expertise and professional identity formation. A second limitation is that this was a single site study conducted at a private not-for-profit medical school and with a single class of students, which may limit transferability of the study results. However, the composition of the student population aligns demographically with other medical schools in the U.S. and as such may provide transferable information within similar contexts.

## Conclusions

The results of this study have significance for medical educators engaged in curricular reform efforts that impact how the biomedical sciences are taught. Specifically, the study reveals new insight into how medical students, who have completed two-years of study of the biomedical sciences within an integrated curriculum, perceived the relevance of biomedical science knowledge to the practice of medicine during the first clerkship year of the undergraduate medical curriculum. The findings suggest that students recognized that their biomedical science knowledge contributed to the development of adaptive expertise and professional identity formation. As the goal of medical education is to equip physicians with the requisite biomedical science knowledge to make clinical decisions and practice evidence-based medicine, and the skills and knowledge to effectively communicate with patients and engage them in shared decision-making, the findings herein suggest caution when revising curricula. Our study supports the notion of others that the loss of clinical expertise deeply grounded in biomedical science and an understanding of the pathologic basis of disease may negatively impact the development of adaptive expertise and professional identity formation [50, 51]. Finally, we recommend that future studies identify the contextual factors of the learning environment, including both explicit and tacit elements of the formal, informal, and hidden curriculum, that enable biomedical science knowledge to contribute to these developmental processes so that they can be leveraged rather than lost during curricular reform [52].

## Supplementary information

**Additional file 1.**

## Data Availability

The datasets used and/or analyzed during the current study are available from the corresponding author on reasonable request.
