# Correction to: “It is this very knowledge that makes us doctors”: an applied thematic analysis of how medical students perceive the relevance of biomedical science knowledge to clinical medicine

**DOI:** 10.1186/s12909-020-02371-3

**Published:** 2020-11-13

**Authors:** Bonny L. Dickinson, Kristine Gibson, Kristi VanDerKolk, Jeffrey Greene, Claudia A. Rosu, Deborah D. Navedo, Kirsten A. Porter-Stransky, Lisa E. Graves

**Affiliations:** 1 Department of Biomedical Sciences, Mercer University School of Medicine, Macon, GA USA; 2 Department of Pediatric and Adolescent Medicine, Western Michigan University Homer Stryker M.D. School of Medicine, Kalamazoo, MI USA; 3 Department of Family and Community Medicine, Western Michigan University Homer Stryker M.D. School of Medicine, Kalamazoo, MI USA; 4 Department of Medical Education, Western Michigan University Homer Stryker M.D. School of Medicine, Kalamazoo, MI USA; 5 Center for Interprofessional Studies and Innovation at Massachusetts General Hospital Institute of Health Professions, Boston, MA USA; 6 Massachusetts General Hospital, Learning Laboratory, Massachusetts General Hospital, Boston, MA USA; 7 Department of Biomedical Sciences, Western Michigan University Homer Stryker M.D. School of Medicine, Kalamazoo, MI USA

**Correction to: BMC Med Educ 20, 356 (2020)**

**https://doi.org/10.1186/s12909-020-02251-w**

Following publication of the original article [1], the authors Kristine Gibson, Jeffrey Greene and Lisa E. Graves changed their affiliations as follows:

Kristine Gibson

From: “Department of Family and Community Medicine, Western Michigan University Homer Stryker M.D. School of Medicine, Kalamazoo, MI, USA.“

To: “Department of Pediatric and Adolescent Medicine, Western Michigan University Homer Stryker M.D. School of Medicine, Kalamazoo, MI, USA.”

Jeffrey Greene

From: “Department of Family and Community Medicine, Western Michigan University Homer Stryker M.D. School of Medicine, Kalamazoo, MI, USA.”

To: “Department of Medical Education, Western Michigan University Homer Stryker M.D. School of Medicine, Kalamazoo, MI, USA.”

Lisa E. Graves

From: “Department of Biomedical Sciences, Mercer University School of Medicine, Macon, GA, USA”

To:” Department of Family and Community Medicine, Western Michigan University Homer Stryker M.D. School of Medicine, Kalamazoo, MI, USA.”

The author group has been updated above.
