# Supplementary material for: “It is this very knowledge that makes us doctors”: an applied thematic analysis of how medical students perceive the relevance of biomedical science knowledge to clinical medicine
Source: BMC Med Educ. 2020 Oct 12;20:356. doi: 10.1186/s12909-020-02251-w (PMC7552568; doi:10.1186/s12909-020-02251-w)
Supplement: Supplementary file 1 — Additional file 1. [file 12909_2020_2251_MOESM1_ESM.docx]

**Reflective writing assignment – Family and community medicine and pediatrics clerkship rotations**

**Pre-clerkship assignment - completed by the end of the preparatory week**

In the preparatory week of the family medicine and pediatric rotations, students were asked to complete the following written assignment: Please write a cogent argument in paragraph form (200-400 words) to address the following question: How is biomedical science knowledge relevant to clinical medicine?

**Mid-clerkship assignment - completed by the end of the clerkship rotation**

Every patient provides a link between biomedical science and clinical care. In your next clerkship, identify one patient with whom to explore this link through a reflective writing exercise. There are no right or wrong answers in reflective writing. It is important to describe how you have reached your conclusions.

Please write a single paragraph for each of the following four sections:

1. Identify a patient you have seen whose illness or disease raised questions for you that required you to access and/or expand your basic science knowledge. Briefly describe your encounter with this patient.
2. Think about the patient’s illness or disease process through a basic science lens by accessing, exploring, and extending your biomedical science knowledge.
   1. List the 3-5 basic science learning objectives related to the care of this patient. Identify appropriate learning resources that will enable you to actively acquire *new* biomedical science knowledge to enrich and expand your existing knowledge.

In a separate paragraph, address:

- 1. How you and/or your preceptor identified biomedical science links related to this patient encounter.
  2. Explain why and how you selected the learning resources to address the basic science learning objectives.
  3. What basic science links did you discover?

1. Having now gone through the process of viewing a patient through a basic science lens and deliberately linking basic science knowledge to this patient, address the following:
   1. How did this process allow you to develop a deeper meaning and understanding of the illness or disease process?
   2. In what ways is this process a useful step in clinical care?
   3. Describe how you will continue to apply this process with other patients.
   4. What new biomedical science links did you identify with other patients?
   5. How did you share this process with your peers, team, or preceptor? What was their reaction(s)?
2. Describe your reflection on the meaning and implications of this reflective experience and consider how it may impact your future clinical practice. Address the following:
   1. Describe how you would consider using this process of viewing a patient and/or clinical problem through a basic science lens in the future. For example, will you do or say something differently the next time a similar situation is confronted? Will you adopt a new attitude or a change in thinking that may influences your future actions?
   2. Review the preparatory week assignment you submitted. Has your argument changed? Why or why not?
